# A rapid and reliable chromosome analysis method for products of conception using interphase nuclei

**DOI:** 10.1002/mgg3.381

**Published:** 2018-03-24

**Authors:** Ramesh Babu, Daniel L. Van Dyke, Saurabh Bhattacharya, Vaithilingam G. Dev, Mingya Liu, Minjae Kwon, Guangyu Gu, Prasad Koduru, Nagesh Rao, Cynthia Williamson, Ernesto Fuentes, Sarah Fuentes, Stephen Papa, Srikanthi Kopuri, Vandana Lal

**Affiliations:** ^1^ Department of Research and Development InteGen LLC Orlando FL USA; ^2^ Department of Laboratory Medicine and Pathology Mayo Clinic Rochester MN USA; ^3^ Departments of Cytogenetics and Administration Dr. Lal PathLabs Ltd. New Delhi India; ^4^ Department of Clinical Cytogenetics Genetics Associates Inc. Nashville TN USA; ^5^ Department of Pathology UT Southwestern Medical Center Dallas TX USA; ^6^ Department of Pathology and Laboratory Medicine David Geffen UCLA School of Medicine Los Angeles CA USA; ^7^ Dr. Lal PathLabs Ltd. New Delhi India

**Keywords:** abortion, fluorescence, in situ hybridization, karyotype, spontaneous

## Abstract

**Background:**

Karyotype determination has a central role in the genetic workup of pregnancy loss, as aneuploidy (trisomy and monosomy) and polyploidy (triploidy and tetraploidy) are the cause in at least 50% of first trimester, 25% of second trimester, and 11% of third trimester miscarriages. There are several limitations with the current approaches of obtaining a karyotype using traditional cytogenetics, fluorescence in situ hybridization with a limited number of probes, and chromosomal microarray. These include culture failure, incomplete results, lower sensitivity, and longer reporting time.

**Methods:**

To overcome current limitations, a novel molecular assay is developed with a Standard Resolution Interphase Chromosome Profiling probe set which is a variation of the recently developed High Resolution probe set. It generates a molecular karyotype that can detect all major changes commonly associated with pregnancy loss. Initial familiarization of signal patterns from the probe set was used, followed by validation of the method using 83 samples from miscarriages in a blind study from three different laboratories. Finally, the clinical utility of the method was tested on 291 clinical samples in two commercial reference laboratory settings on two different continents.

**Results:**

The new molecular approach not only identified all the chromosome changes observed by current methods, but also significantly improved abnormality detection by characterizing derivative chromosomes and finding subtle subtelomeric rearrangements, balanced and unbalanced. All Robertsonian translocations were also detected. The abnormality rate was 54% on clinical samples from commercial laboratory 1 and 63% from laboratory 2.

**Conclusion:**

The attributes of this method make it an ideal choice for the genetic workup of miscarriages, namely (1) near 100% successful results, (2) greater sensitivity than conventional chromosome analysis or FISH panels, (3) rapid reporting time, and (4) favorable comparisons with chromosomal microarray.

## 1 INTRODUCTION

Karyotype determination has a central role in the genetic workup of pregnancy loss, as aneuploidy (trisomy and monosomy) and polyploidy (triploidy and tetraploidy) are the cause in at least 50% of first trimester losses, 25% of second trimester, and 11% of third trimester miscarriages (Van Dyke & Wiktor, 2002). The three primary methods used to obtain this information are conventional chromosome analysis, targeted FISH, and more recently chromosomal microarray (Wang et al., 2017). Each of these techniques has their advantages and disadvantages. While the conventional approach covers the whole genome, the cell culture failure rate generally exceeds 20%, and 13%–20% of the successful cell cultures yield only maternal cells (Lathi et al., 2014; Murugappan, Gustinl, & Lathi, 2014; Shearer, Thorland, Carlson, Jalal, & Ketterling, 2011). In addition, the time taken to release the chromosome analysis report is typically two‐four weeks. Targeted FISH analysis generally covers no more than chromosomes 13, 16, 18, 21, 22, X, and Y, and therefore provides incomplete information. Also, reporting time for targeted FISH ranges from 5 to 14 days and it does not detect balanced or unbalanced chromosome rearrangements (Shearer et al., 2011). Chromosomal microarray (CMA), especially SNP‐based microarray, is more reliable than conventional chromosome analysis and more informative than targeted FISH, however, it cannot identify the chromosomal basis of deletions or duplications including Robertsonian translocations, it can be troubled by polyploidy, mosaicism, and maternal cell contamination, and CMA reporting time is typically a week or longer (Caramins et al., 2011; Wang et al., 2017).

We recently developed and validated a novel technology, Interphase Chromosome Profiling (ICP), for high resolution chromosome analysis of hematologic malignancies (Babu et al., 2018). Here, we describe development and validation of ICP and a Standard Resolution probe set variation of ICP that can be employed to karyotype products of conception (POC) samples. The major attributes of the ICP technology, including nearly 100% success rate, higher sensitivity than traditional karyotype and FISH, and fast reporting time, were validated and evaluated in a commercial clinical service laboratory setting.

## 2 MATERIALS AND METHODS

This study was determined to be exempt from IRB review by the Mayo Clinic Institutional Review Board.

**Figure 1:**
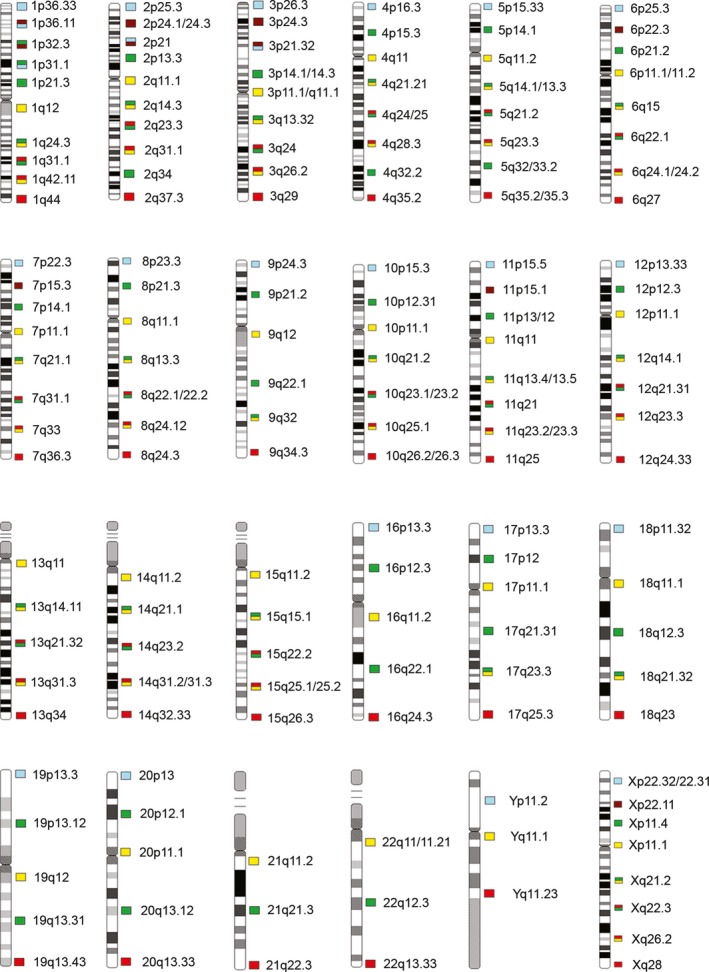
High Resolution Interphase Chromosome Profiling Ideogram. Illustration of the High Resolution ICP ideogram at approximately 600 band level showing each color band and its corresponding ISCN band designation. Reprinted from Babu R, Van Dyke DL, Dev VG et al., entitled Interphase Chromosome Profiling: A Method for Conventional Banded Chromosome Analysis Using Interphase Nuclei. Arch Pathol Lab Med. 2018;142(2):213‐228, with permission from Archives of Pathology & Laboratory Medicine. Copyright 2018 College of American Pathologists.

**Figure 2:**
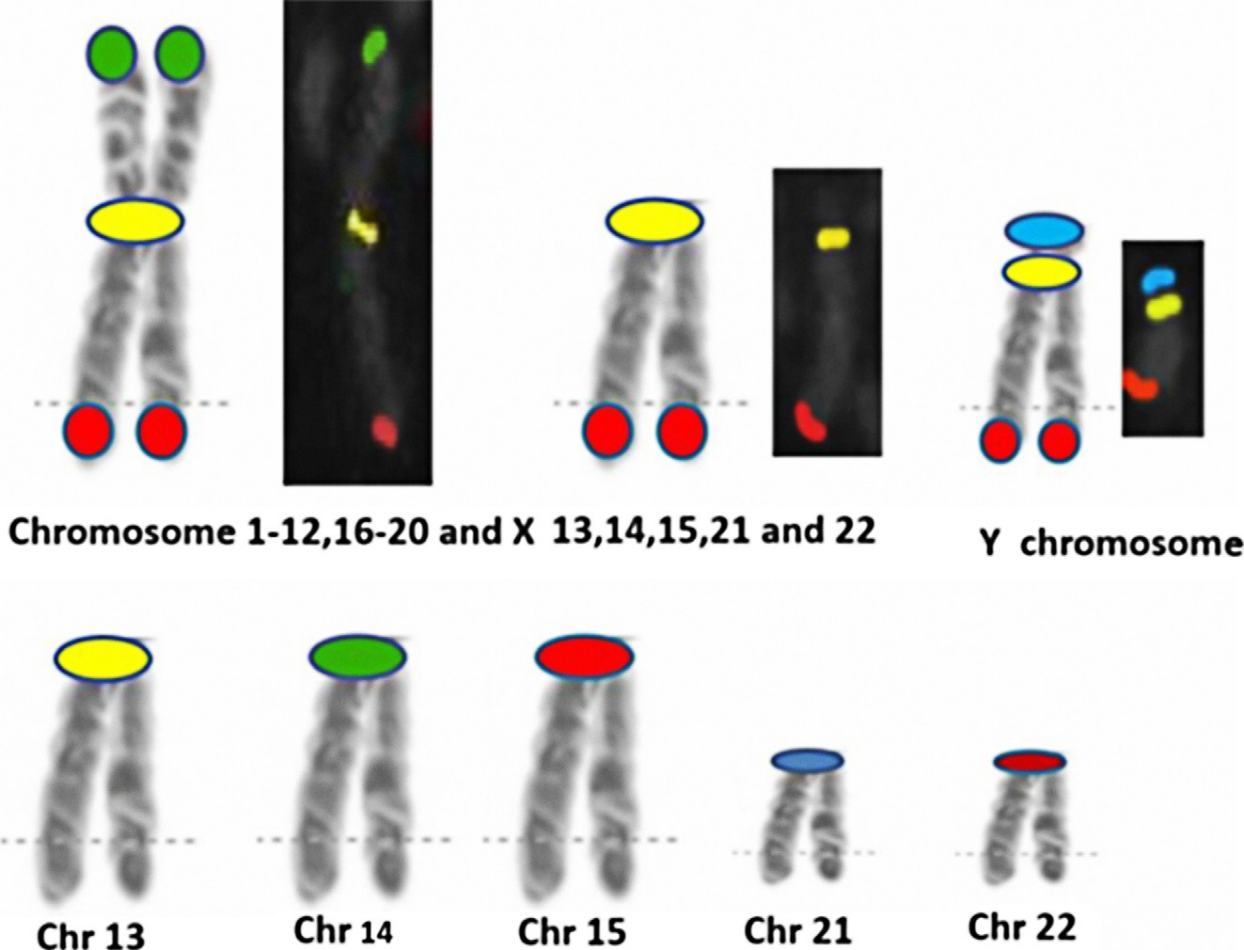
Standard Resolution Interphase Chromosome Profiling design. The top row illustrates a typical metacentric chromosome with green and red signals in the distal short and long arms, respectively, and a yellow pericentromeric signal. In the middle is a typical acrocentric chromosome with a yellow pericentromeric signal and a red distal long arm signal. The acrocentric chromosomes have no short arm signal. The Y chromosome (far right) short arm has an aqua signal, and the pericentromeric and distal long arm signals are the same as in other autosomes and X chromosome. The bottom row illustrates the distinct pericentromeric signal for each acrocentric chromosome, which is present in a mix that is used in a separate hybridization to identify a Robertsonian translocation. The chromosome 13 signal is yellow, 14 is green, 15 is red, 21 is aqua, and 22 is far red. No other regions of acrocentric chromosomes are targeted in this mix

The High Resolution ICP design (InteGen, Orlando, FL) is based on the equidistant concept of placing FISH probes along the entire length of the chromosome (Figure 1), as originally developed for evaluation of hematologic malignancies (Babu et al., 2018). Briefly, each chromosome arm includes at least one (18p and Yp) and up to six (2q, 4q, and 5q) hybridization sites, each assigned to a specific chromosome band. Subtelomeric and pericentromeric sequences are assigned a pure color (aqua, yellow, and red for pter, centromere, and qter, respectively), and interstitial bands are assigned either a pure (far red or green) or a hybrid (fusion) color. Each chromosome is studied individually, with the results compiled into a composite karyotype. This configuration provides the equivalent of a 600‐band resolution karyotype (McGowan‐Jordan, Simons, & Schmid, 2016) and facilitates the identification of copy number changes of whole chromosomes as well as balanced and unbalanced chromosome rearrangements. A Standard Resolution ICP probe set (InteGen, Orlando FL) was developed (Figure 2) that targets only the subtelomere and pericentromeric regions, since the abnormalities commonly encountered in POC samples, such as trisomy and unbalanced translocations, can be easily detected with this simplified design. Chromosome breakpoints are typically shown as question‐marks because the High Resolution ICP was not employed for the POC study. An Acrocentric ICP probe set (InteGen, Orlando FL) was designed specifically to detect Robertsonian translocations (Figure 2), wherein each acrocentric chromosome's pericentromeric area is targeted, and a mixture of these five targets is used in a separate analysis.

Individual chromosome hybridizations were done on four microscope slides, with six areas of hybridization on each slide, following standard FISH protocols (Pinkel, Straume, & Gray, 1986; Trask & Pinkel, 1990) with minor adjustments. Roughly 20 ng of probe was used for each chromosomal target which were hybridized overnight in a 10 mm area under a round coverslip. Posthybridization washing conditions included two minutes in 0.4× SSC/0.3% NP‐40 at 69°C followed by one minute in 2× SSC/0.1% NP‐40 at room temperature. DAPI counterstaining was omitted. Appropriate filter sets from Semrock (Rochester NY) were used to detect fluorophores DEAC (aqua), Fluorescein‐12 (green), Cyanine555 (yellow), Cyanine647 (far red), and CF594 (red). Initial scanning to place cells in the correct plane was done using the filter for Cyanine555. A minimum of 20 interphase cells were analyzed for each chromosome, to mimic the usual guidelines of metaphase analysis to identify mosaicism; here, mosaicism is generally accepted if seen in at least four of 20 interphase cells.

Each autosome was analyzed separately, and the X and Y chromosomes were analyzed together. For the assessment of Robertsonian translocations, the Acrocentric ICP probe set was employed. Similar to typical FISH studies, a normal cut‐off was established for the study and should be set by each performing laboratory (Wiktor et al., 2006). For this study, the normal cut‐off was set at 20% for any two pericentromeric probes to colocalize by random chance or by satellite association. Any juxtaposition of two signals in >20% of interphase nuclei was considered abnormal.

Following a familiarization step, the study consisted of two major stages. In stage 1, three separate laboratories, including two clinical laboratories and the laboratory that developed the technology, validated the Standard ICP protocol on POC samples that had been previously cultured, harvested, and analyzed using traditional cytogenetic methodology. Initially, four samples with a Robertsonian translocation were studied nonblindly to familiarize the signal patterns suggestive of a juxtaposition of two acrocentric chromosome pericentromeric regions. Several known abnormalities, including trisomy, monosomy, triploidy, and tetraploidy, were also studied to recognize signal patterns generated with the Standard design. Next, the laboratory that developed the technology received 43 samples of POC material from three participating laboratories for a blind study (Table 1). Simultaneously, two commercial laboratories on two different continents validated the technology in a blind study. Laboratory 1 studied five samples (data not shown), since they had already validated High Resolution ICP for hematologic malignancies, and the second laboratory studied 40 samples (Table 2).

**Table 1:** Initial validation using standard resolution interphase chromosome profiling

Specimen no.	Cytogenetic result	ICP Result	Concordant
1–10, 12, 18, 26, 28, 30, 33, 35, 38–41	Normal	Normal	Yes
11	Trisomy 16	Trisomy 16	Yes
13	Monosomy X	Monosomy X,del(17q)	Yes, and new changes
14	Trisomy 18	Trisomy 18	Yes
15	Triploid	Triploid	Yes
16	Normal female	Normal male/female	Discordant
17	Normal female	Trisomy 22 (6 cells)	Discordant
19, 21, 23, 25	+21,der t(14;21)	+21,der t(14;21)	Yes
20	+15,der(13;15)	+15,der(13;15)	Yes
22	+13,der(13;14)	+13,der(13;14)	Yes
24	+14,t(13;22)	+14	Discordant
27, 31	Male trisomy 16	Normal female	Discordant
29, 32	Trisomy 10	Trisomy 10	Yes
34	Tetraploid	Tetraploid	Yes
36, 42	Trisomy 21	Trisomy 21	Yes
37, 43	Culture Failure	Normal	New result

ICP, interphase chromosome profiling.

**Table 2:** Initial commercial laboratory validation using standard resolution interphase chromosome profiling

Specimen no.	Cytogenetic result	ICP result	Concordant
1–10	Normal	Normal	Yes
11, 24, 27	Triploid	Triploid	Yes
12, 20, 21, 29, 32, 34, 35, 39, 40	Monosomy X	Monosomy X	Yes
13, 28	Trisomy 13	Trisomy 13	Yes
14, 16, 18, 22, 23, 26, 37, 38	Tetraploid	Tetraploid	Yes
15	Monosomy X	Monosomy X,del(Xq)	Concordant, new change
17	Tetraploid (mosaic 35%)	Tetraploid (mosaic)	Yes
19, 30	Trisomy 18	Trisomy 18	Yes
25, 33, 36	Trisomy 21	Trisomy 21	Yes
31	Trisomy 21	Trisomy 21, t(2;10)	Concordant, new change

ICP, interphase chromosome profiling.

**Figure 3:**
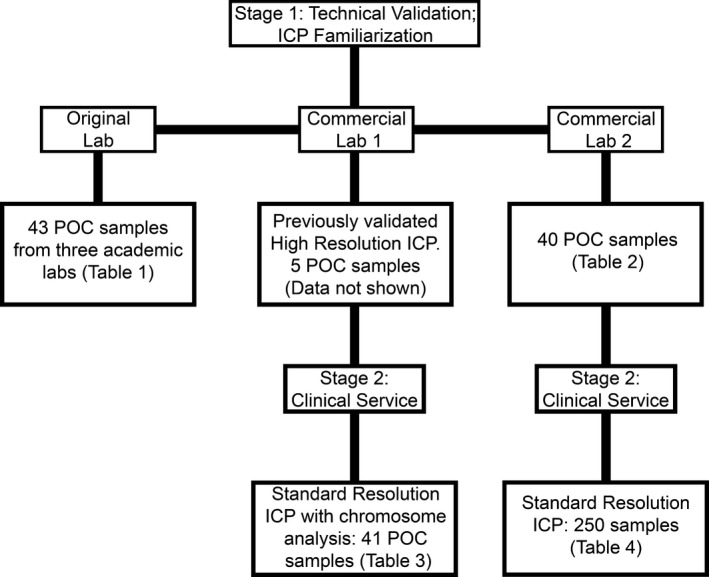
A flow diagram depicting the validation steps

In stage 2, each commercial laboratory tested clinical samples using the Standard Resolution ICP probe set. The ICP protocol followed by each laboratory was identical, and the analysis was done as discussed above. Cell culture time was limited to overnight, and there was no mitotic arrest. Standard hypotonic and fixation protocols were used. Laboratory 1 tested 41 samples (Table 3), and Laboratory 2 tested 250 samples (Table 4). Laboratory 1 also employed G‐banded analysis concurrently on these 41 samples. Laboratory 2, having previously validated the ICP methods, did not undertake concurrent conventional chromosome analyses. A flow diagram depicting all the steps described above is shown in Figure 3.

**Table 3:** Stage 2 clinical data from commercial laboratory 1 using standard resolution interphase chromosome profiling

Sp. no.	Cytogenetic result	ICP result	Concordant
1	icp.46,XY,der(13;14)(q10;q10),+14	icp.46,XY,der(13;14)(q10;q10),+14	Yes
2, 3, 8, 24, 25	45,X	icp.45,X	Yes
4	46,XY,del(4)(p15.2p16.3)	icp.46,XY,del(4)(pter)	Yes
5	47,XX,+15	icp.47,XX,+15	Yes
6, 20	69,XXX	icp.69,XXX	Yes
7	47, XY, +13	icp.47, XY, +13	Yes
9, 16	47,XX,+22	icp.47,XX,+22	Yes
10, 12, 18, 26, 29–31, 33, 35, 37, 41	46, XX	icp.46,XX	Yes
11, 15, 17, 19, 22, 27, 28, 36	46,XY	icp.46,XY	Yes
13	46,XX,add(18)(q23)	icp.46,XX,der(18)t(7;18)(p?;q?)	Yes and identified change
14	47,XX,+18	icp.47,XX,+18	Yes
21, 34	47,XX,+21	icp.47,XX,+21	Yes
23	47,XY,+16	icp.47,XY,+16	Yes
32	47,XY,t(2;11)(q33;q13),+22	icp.47,XY,t(2;11)(q?;q?),+22	Yes
38	47, XY, +21	icp.47, XY, +21	Yes
39	69,XXY	icp.69,XXY	Yes
40	47,XY, +20	icp.47,XY, +20	Yes

ICP, interphase chromosome profiling; Sp., specimen.

**Table 4:** Stage 2 clinical data from commercial laboratory 2 using standard resolution interphase chromosome profiling

Specimen no.	No. female samples	No. male samples	ICP result
1–100	57	43	icp.46,XX or XY
101–107	7	0	icp.45,X
108–129	15	7	icp.47,XX or XY,+16
130–199	29	41	icp.47,XX or XY,+21
200, 201	2	0	icp.92,XXYY
202	1	0	icp.92,XXXX/46,XX
203–213	3	8	icp.47,XX or XY,+13
214–218	4	1	icp.47,XX or XY,+18
219	1	0	icp.45,XY,−16
220, 221	1	1	icp.47,XX or XY,+14
222, 223	2	0	icp.47,XX,+15
224, 225	2	0	icp.47,XX,+22
226	1	0	icp.47,XY,+4
227	1	0	icp.47,XX,+19
228	1	0	icp.47,XX,+20
229	1	0	icp.48,XX,+7,+16
230	1	0	icp.48,XX,+13,+16
231	1	0	icp.48,XXY,+15
232	1	0	icp.48,XXY,+16
233, 234	1	1	icp.47,XXX or XXY
235	1	0	icp.46,XX,t(2;18)(p?;q?)
236	1	0	icp.46,XX,t(12;14)(p?;q?)
237	1	0	icp.46,XY,der(11)t(1;11)(q?;p?)
238, 239	2	0	icp.46,XY,del(9)(p?)
240, 241	2	0	icp.46,XY,del(16)(q?)
242	1	0	icp.47,XX,der(14;21),+21
243	1	0	icp.46,XX,der(4)t(4;21)(q?;q?)
244	1	0	icp.46,XX,del(10)(p?)
245–247	2	1	icp.69, or XXY
248	1	0	icp.47,XY,+8
249, 250	1	1	icp.92,XXXX or XXYY

ICP, interphase chromosome profiling.

Specimens fixed with formalin and embedded in paraffin (FFPE) were not employed for this study, but ICP has been validated on FFPE material (unpublished data).

## 3 RESULTS

**Figure 4:**
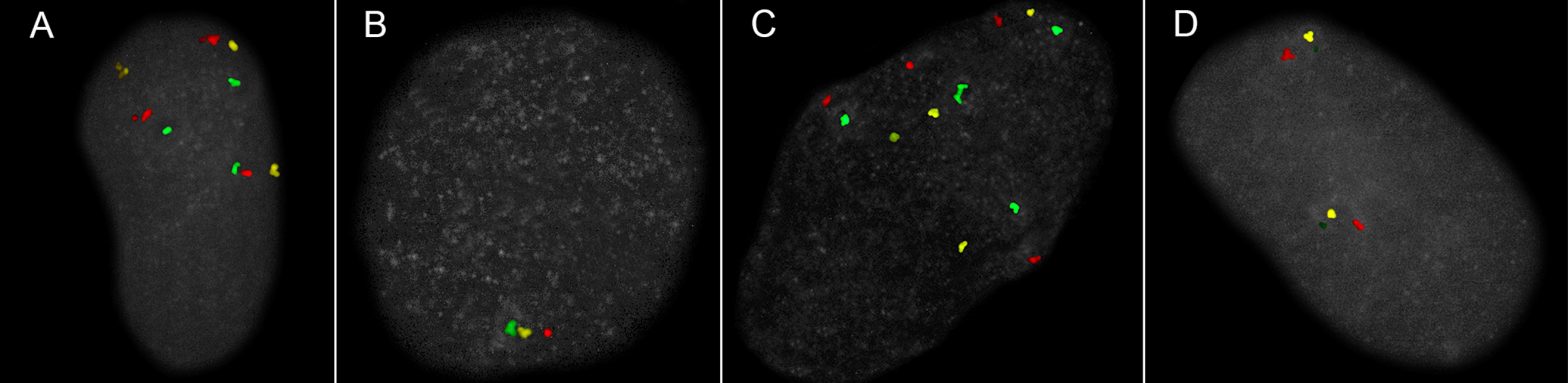
Examples of numerical abnormalities. Image (a) is a trisomy with three green, yellow, and red signals. Image (b) is a monosomy with only one green, yellow, and red signal. Image (c) is tetrasomy, with four of each signal. A similar pattern for the other autosomes would indicate a tetraploid conceptus. Image (d) is a typical normal diploid signal pattern

The first stage in the clinical validation employed 83 blinded samples with known cytogenetic results identified by conventional cytogenetics, FISH, or both (Tables 1 and 2). These samples included trisomy, monosomy, triploid, tetraploid, and balanced and unbalanced Robertsonian translocations (Tables 1 and 2, Figure 4). There were successful ICP results for both of the failed cytogenetic cases (cases 37 and 43, Table 1, Figure 4). In almost all cases, there was complete concordance with the conventional chromosome analysis.

The second stage in the clinical validation tested the clinical utility of Standard Resolution ICP in another set of 291 clinical samples, which were referred for chromosome testing in the commercial reference laboratory setting in two different laboratories. Laboratory 1 tested 41 samples and all of the samples had an ICP result (100%) with a 54% abnormality rate. There was 100% concordance with conventional cytogenetics on these 41 samples (Table 3). Laboratory 2 tested 250 samples with 100% successful ICP results and an abnormality rate of 63% (Table 4). The average turnaround time was <48 hr in both commercial laboratories.

**Figure 5:**
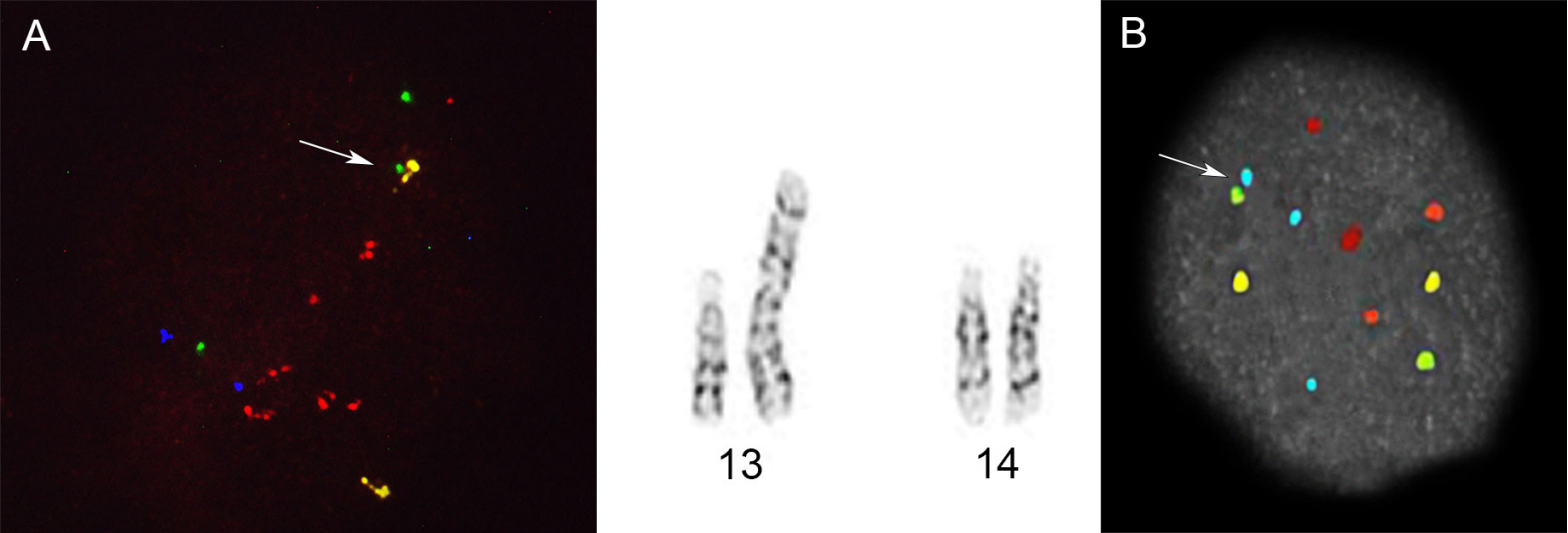
Robertsonian translocations. Using the Acrocentric ICP probe set (see Figure 2), Figure 4(a) has two red, two aqua and two far red signals indicating normal pairs of chromosomes 15, 21, and 22. There is one free yellow signal and two free green signals for chromosomes 13 and 14. There is also one consistently fused yellow and green signal, representing the unbalanced 13;14 translocation in case 1 (Table 3). Trisomy 14 is evident by the G‐banded chromosomes displayed to the right. Shown in the upper left of image Figure 4(b) is the consistent fusion of a green chromosome 14 signal and a third aqua chromosome 21 signal from case 242 (Table 4), indicating the translocation trisomy 21

Aneuploidy that is generally not detected with commonly used FISH panels, but which was observed in the ICP studies, included 48 cases with trisomy 4, 7, 8, 14, 15, 19, or 20. ICP found 21 structural abnormalities, including autosomal balanced translocations and Robertsonian translocations, some of which FISH or CMA would have failed to detect or correctly characterize. Three rearrangements discovered by ICP in the initial phase of the study were not identified by conventional chromosome analysis, and one abnormality found by conventional chromosome analysis was not recognized by ICP (see next paragraph). Application of the Acrocentric ICP probe set was used in the evaluation of acrocentric trisomies (Figures 2 and 5). ICP and Acrocentric ICP identified eight Robertsonian translocations, four apparently balanced reciprocal translocations, and nine unbalanced rearrangements including derivative chromosomes and terminal deletions.

**Figure 6:**
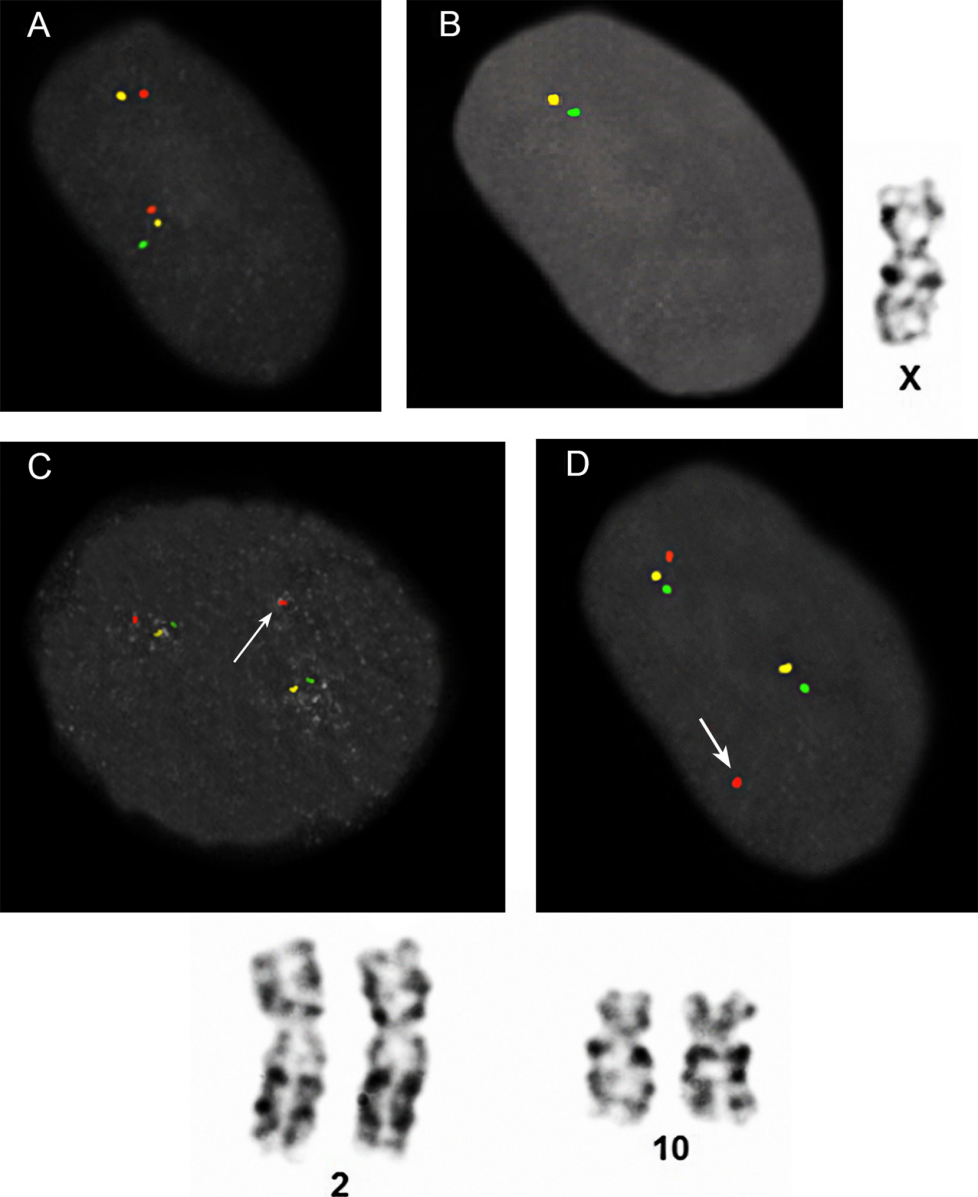
Examples of deletions and a balanced translocation. (a) Case 238, Table 4: short arm deletion of one chromosome 9 illustrated by the absence of green signal on the chromosome on the top (left panel); (b) Case 15, Table 2: deletion of the long arm telomere region on the only X chromosome by the absence of the red signal (middle panel); in the far right is a metaphase chromosome X from the same case presented in the middle panel. The deletion was not evident by G‐banding. (c and d). Case 31, Table 2: a balanced translocation between chromosomes 2 and 10. Displacement of the red signals from the long arms of chromosomes 2 (c) and 10 (d) are indicated by the arrows. Partial karyotypes of chromosomes 2 and 10 from the same case in (c) and (d) are shown below. The translocation was not evident by G‐banding, and was likely inherited from a balanced carrier parent

The results of cases 32 (Table 3), 235 (Table 4), and 236 (Table 4) in this study demonstrate detection of balanced rearrangements using ICP. One case in the validation study (case 31, Table 2, Figure 6b–d) demonstrated that a subtelomeric balanced rearrangement is readily detected by ICP, while it can be missed by conventional chromosome analysis.

**Figure 7:**
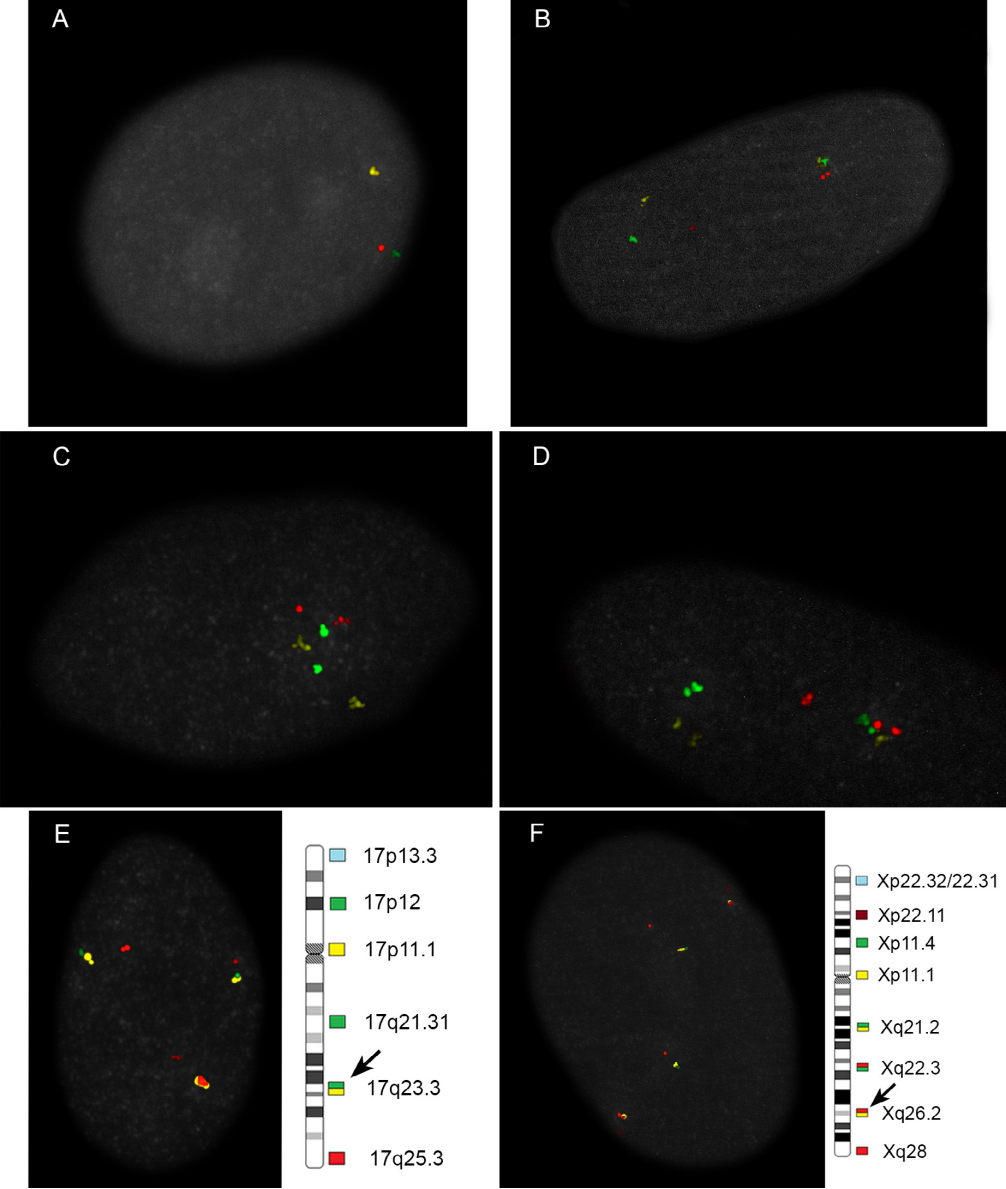
Mosaic monosomy X and 17q deletion. Regarding case 13 (Table 1), image (a) shows the monosomy X (compare with Figure 3a). Image (b) shows the 17q deletion with the constant dim red signal on one chromosome 17. Images (c) and (d) illustrate normal chromosome 17 pairs from a different case (c) and a different cell from case 13 (d). (e) Chromosome X and 17 hybridized together with unique tags on each chromosome showing monosomy X and 17q deletion. Green/yellow tag (at band 17q23.3) tracks chromosome 17 q telomere and red/yellow (at band Xq26.2) tag tracks X chromosome long arm telomere. (f) A normal cell with same tags as in (e). Tag locations are shown by solid arrows on the corresponding high‐resolution chromosome ideograms

In five cases, an abnormality was identified using ICP that was not described in the conventional chromosome analysis. Case 13 (Table 1, Figure 7) was mosaic (75%) monosomy X, and ICP detected a subtle distal 17q deletion in the monosomy X cell population. In case 15 (Table 2, Figure 6a), monosomy X was classified by both methods, and ICP identified a distal Xq deletion. In case 31 (Table 2, Figure 6b,c), trisomy 21 was found by both methods, and ICP recognized an apparently balanced 2;10 translocation. In case 17 (Table 1), the karyotype was uniformly normal female, whereas ICP identified a mixture of normal female and trisomy 22 male. Seven of the eight Robertsonian translocations were detected by both methods. Case 24 (Table 1) had trisomy 14 identified by both methods in addition to a balanced 13;22 Robertsonian translocation that was not identified in the initial ICP evaluation. However, it was confirmed on re‐evaluation. This discrepancy was likely a “learning curve” oversight during the early stages of the validation.

**Figure 8:**
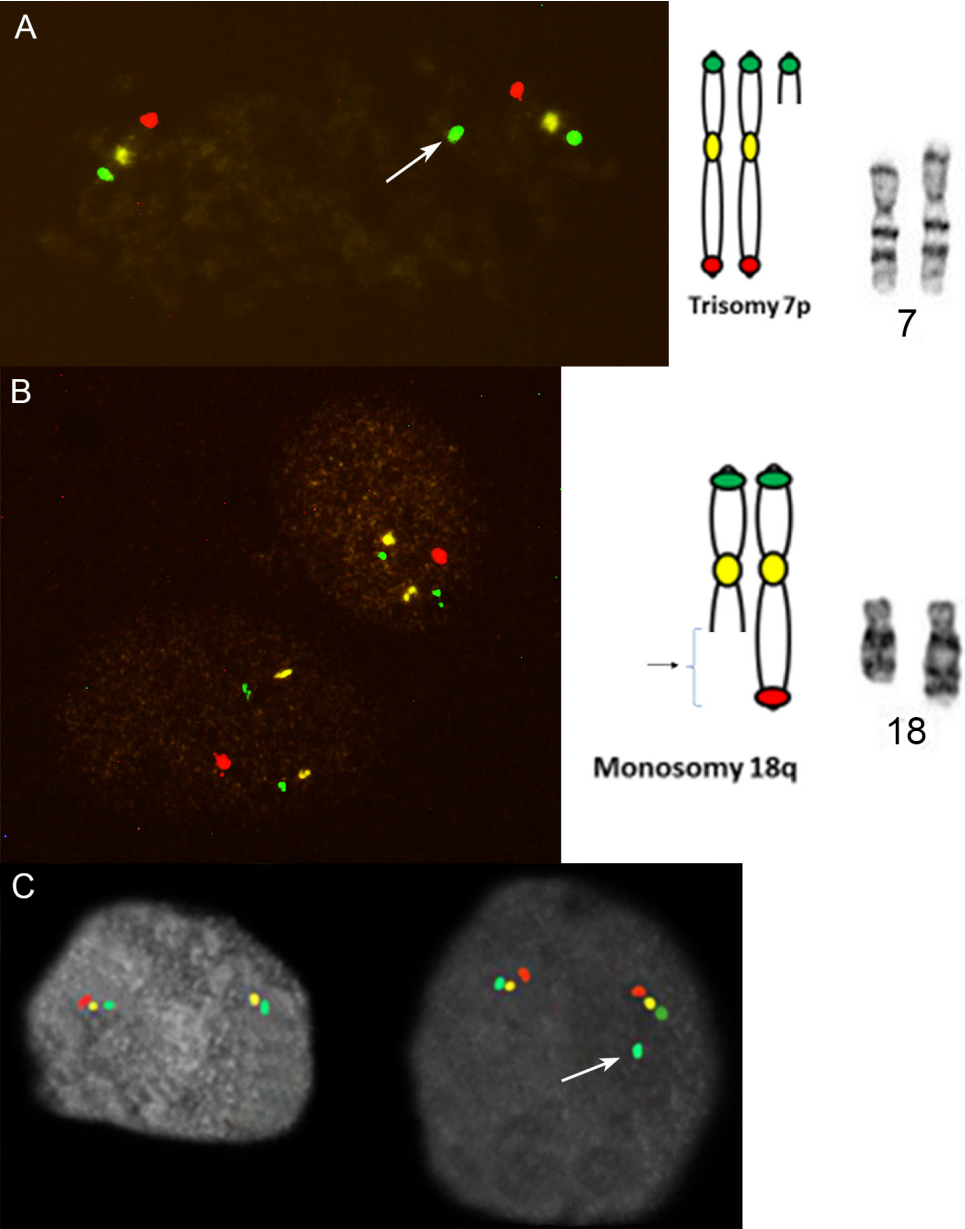
Characterization of a cytogenetically undefined “add” material on 18q. Patient 13, Table 3 was originally described as an add(18), and using ICP was further defined as an unbalanced translocation between chromosomes 7 and 18 with duplication 7p and deletion 18q. (a) Chromosome 7 showing the extra green band (b). Loss of one red signal on chromosome 18. (c). Case 237, Table 4, here ICP characterized an unbalanced translocation between chromosomes 1 and 11 showing loss of red signal on chromosome 11 (left) and gain of green signal from chromosome 1 indicated by arrow (right)

**Figure 9:**
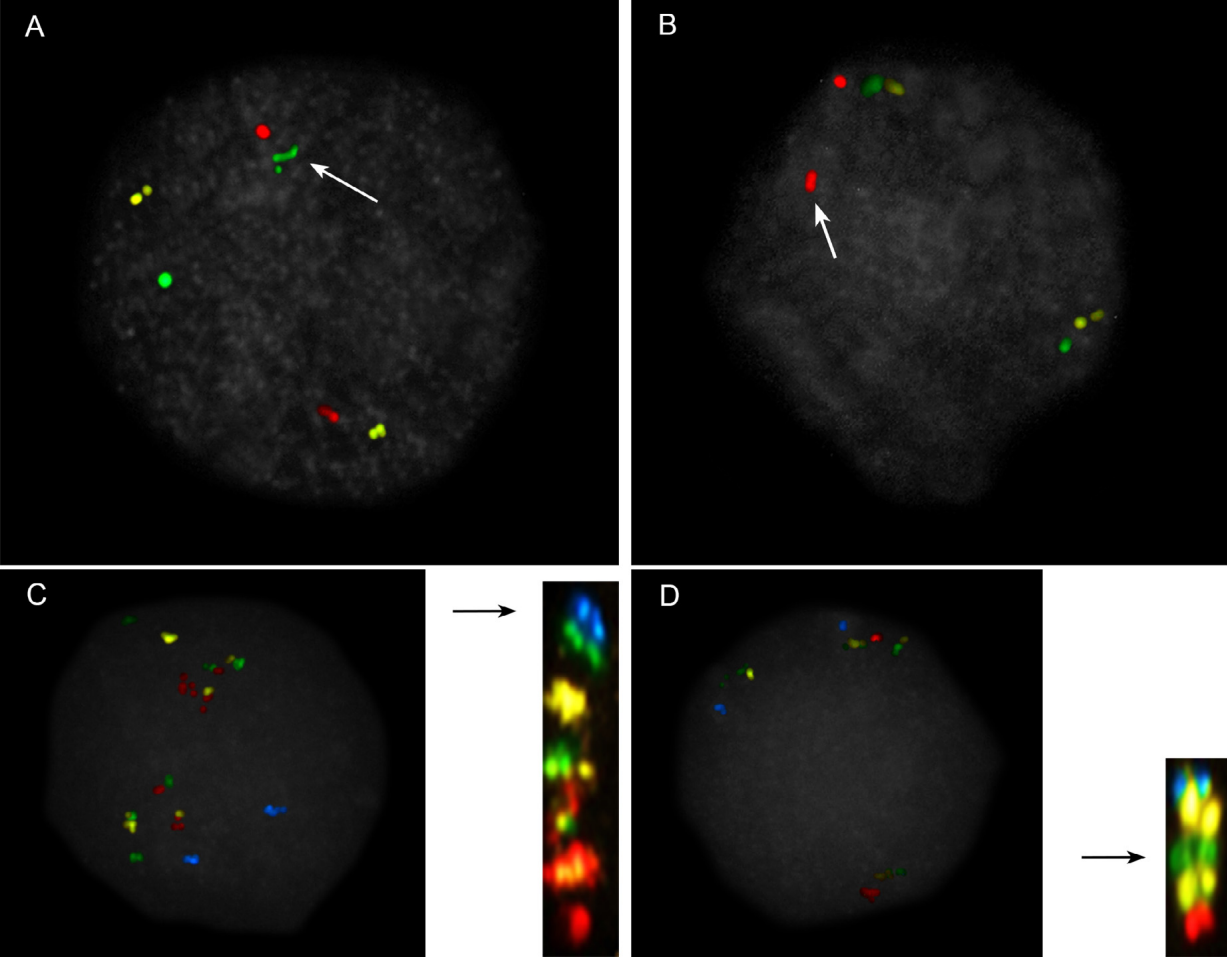
Precise determination of translocation breakpoints. Case 18 from the recent high resolution ICP study in leukemia (Babu et al., 2018): (a) Initial breakpoint assignment between centromere and the short arm telomere on chromosome 12. (b) Initial breakpoint assignment between centromere and the long arm telomere on chromosome 18. (c) and (d). Breakpoint clarification as 12p13 and 18q12, respectively, using the High Resolution ICP probe set. Arrows on the reference metaphase chromosomes in the middle point out the breakpoints

Other structural abnormalities included two cases with 9p deletion (cases 238 and 239, Table 4, Figure 5), two cases with 16q deletion (cases 240 and 241, Table 4), one case with 10p deletion (case 244, Table 4), one case with 4p deletion (case 4, Table 3) and three balanced translocations (case 32, Table 3 and cases 235 and 236, Table 4). In case 13 (Table 3, Figure 8a,b), ICP characterized the “add” material as an unbalanced 7;18 translocation. In two other cases (case 243, Table 4 and case 237, Table 4, Figure 8c), ICP identified the derivative chromosomes as an unbalanced 4;21 translocation and an unbalanced 1;11 translocation, respectively. A bone marrow sample from the previously published leukemia study (Babu et al., 2018) with an apparently balanced 12;18 translocation illustrates breakpoint assignment using the High Resolution ICP probe set (Figure 9).

Maternal cell contamination (MCC) is a well‐recognized problem in POC cultures. Traditional chromosome studies detected only a normal female karyotype in case 16 (Table 1), however, a few normal male cells were observed by ICP. This testing was done on cell pellets from the conventional cytogenetics monolayer cell culture. Likewise, in case 17 (Table 1) conventional chromosome analysis found only a normal female karyotype (long‐term cell culture), whereas ICP identified trisomy 22. In two other instances (case 27 and 31, Table 1), a male trisomy 16 karyotype was observed, whereas in ICP all cells showed an XX signal pattern – an internal review left open the possibility of a specimen mix‐up error at the originating laboratory.

## 4 DISCUSSION

The clinical management of patients with miscarriages, including genetic counseling regarding recurrence risk for next pregnancy, is often influenced by karyotype findings. Thus, obtaining such information in a fast, accurate, and highly reliable manner is critical. Although other molecular technologies (FISH, CMA, and sequencing) are useful in detecting numerical abnormalities such as trisomy and monosomy, specific detection of balanced and unbalanced structural abnormalities, aneuploidy, and tetraploidy may be problematic depending on the methods employed. In addition, detection of Robertsonian translocations can only be accomplished currently by conventional cytogenetics.

The overall abnormality rate for the clinical samples was 54% in Laboratory 1 and 63% in Laboratory 2, which is comparable with conventional cytogenetic studies when cell culture is successful. The culture failure and MCC rates for POC samples using conventional cytogenetics are high even in the best quality‐controlled laboratories. Culture failure occurs in 15%–20% of attempted studies, and another 20% or more represent maternal‐derived uterine contents, depending to some extent on the referral source (Shearer et al., 2011). For the culture failures, focused FISH studies using a limited number of probes, covering only 5–7 chromosomes, identify numerical abnormalities of those specific chromosomes but no structural abnormalities. Using the ICP method, two cases of cell culture failure (Table 1, cases 37 and 43) were identified as chromosomally normal. Thus, the Standard Resolution ICP probe set can be used to describe a molecular karyotype by analysis of interphase nuclei without dependence on monolayer cell culture or the use of mitotic arrest. In a typical clinical reference or academic laboratory setting, the ICP method can also yield a full karyotype result as quickly as a 5–7 probe FISH panel result.

There were 154 cases of numerical abnormalities and 21 cases of structural abnormalities. Trisomies observed for autosomes 4, 7, 8, 14, 15, 16, 19, 20, and 22 would have been missed by most laboratories using the typical five‐probe (13, 18, 21, X, and Y) FISH assay. Some laboratories include probes for chromosomes 16 and 22, yet they still would have missed the other seven trisomies detected by ICP in this study. ICP also found balanced and unbalanced Robertsonian translocations that would not have been identified by FISH or CMA. Even though most “apparently” balanced translocations do not have any phenotype associated with them, some are associated with a submicroscopic imbalance that could lead to an abnormal phenotype (Baptista et al., 2008; Tabet et al., 2015). The ability to detect such rearrangements was, up until now, only possible by conventional chromosome analysis.

The discovery of deletion 7p and duplication 18q in case 13 (Table 3 and Figure 8a,b) that was previously described as add(18)(q23), illustrates the ability of ICP to better characterize abnormalities that are somewhat cryptic when using conventional chromosome analysis. Similarly, in case 237 (Table 4 and Figure 8c) the 1p deletion and 11q duplication six was detected by ICP. The interstitial 4p deletion (case four, Table 3) illustrates that the Standard Resolution ICP probe set, with subtelomere and centromere probes, is sufficiently sensitive to detect most deletions. There were five cases with “terminal” deletions involving chromosomes 9 (two cases), 16 (two cases), and 10 (one case). Once a structural abnormality is identified using the Standard Resolution ICP method, precise breakpoint determination can be obtained using the High Resolution ICP probe set. Small subtelomeric deletions can also be detected by ICP as demonstrated in case 15 (Table 2, Figure 6b) where the X chromosome had lost the subtelomere region.

Recent studies of pregnancy loss using SNP‐based CMA show its utility in comparison with conventional cytogenetics (Dhillon et al., 2014; Rosenfeld et al., 2015; Sahoo et al., 2017). These studies found a higher success rate and an ability to detect regions of homozygosity suggestive of consanguinity, uniparental disomy, or molar pregnancy. However, challenges remain for CMA related to a failure rate of roughly 8%–10%, the inability to characterize rearrangements or distinguish between primary trisomy and unbalanced Robertsonian translocations, and difficulties with chromosomal mosaicism and interpretation of variants of unknown significance.

However, CMA can identify copy number changes, it does not describe the nature of the abnormality, such as an unbalanced translocation. Two such cases in this study include case 237 (Table 4), in which there was deletion of 11p and duplication of 1q as a result of an unbalanced translocation (Figure 7), and case 243 (Table 4B), with deletion of 4q and duplication of 21q. These cases illustrate the ability of ICP to characterize “add” material on derivative chromosomes.

SNP‐based chromosomal microarray will detect whole genome homozygosity associated with a molar pregnancy as will microsatellite genotyping (Fisher et al., 2014; Furtado et al., 2013), however, when a complete mole is suspected on the basis of morphology, it should be evaluated for p57^KIP2^ immunostaining. Thus, cnLOH is not essential to the evaluation of molar pregnancy.

In comparison to SNP‐based microarray, ICP has a higher success rate and likely faster reporting time and lower cost of equipment, reagents, and personnel effort. In the future, a direct comparison between SNP‐based CMA and ICP would be of interest.

### 4.1 Limitations

Admixture of maternal and fetal cells is a common challenge in cytogenetic studies of pregnancy loss, and efforts to identify the fetal tissues are essential (Murugappan et al., 2014; Shearer et al., 2011). There were instances where the fetal karyotype was identified only by the conventional chromosome analysis (Table 1, cases 27 and 31) and where the fetal karyotype was detected only by the direct ICP analysis (Table 1, cases 16 and 17). Thus, maternal cell contamination remains a preanalytic limitation to cytogenetic testing of products of conception.

Even though the Standard Resolution ICP design used in this study for POC investigation detects gross structural abnormalities including deletions and duplications, most microdeletions, microduplications, and balanced inversions cannot be identified by this technique. These kinds of abnormalities are typically not the cause for miscarriages.

### 4.2 Nomenclature

Until enough experience is gained from studies utilizing this technology and the ISCN committee issues guidance, we propose to add a prefix icp to describe the results generated using the interphase chromosome profiling technology described here. We also use the “composite karyotype” abbreviation “cp” routinely, because the karyotype interpretation is assembled from multiple interphase cells. For example, a sample from a male with an unbalanced Robertsonian translocation between chromosomes 14 and 21 resulting in trisomy 21 could be described as icp.46,XY,der(14;21)(q10;q10),+21[cp20].

## 5 SUMMARY

Standard Resolution ICP appears to be an appropriate tool for first line or reflex testing in the genetic workup of POC samples. Results of this study have confirmed that ICP is (1) highly reliable, (2) more sensitive than the traditional FISH approach using a limited number of probes, (3) capable of detecting both numerical and gross structural aberrations including characterization of “add” material in the derivative chromosomes, and (4) does not require cell culture, which allows a faster reporting time. As with microarray, karyotype analysis, and FISH panels, results of Standard Resolution ICP studies will assist in genetic counseling for recurrence risks of aneuploidy, polyploidy, and balanced and unbalanced chromosome rearrangements.

## CONFLICT OF INTEREST

R.B., E.F., S.F., S.P., and S.K. are employed by InteGen LLC. The remaining authors report no conflicts of interest.

## References

[mgg3381-bib-0001] Babu, R. , Van Dyke, D. L. , Dev, V. G. , Koduru, P. , Rao, N. , Mitter, N. S. ,… Papa, S. (2018). Interphase chromosome profiling (ICP): A method for conventional banded chromosome analysis using interphase nuclei. Archives of Pathology and Laboratory Medicine, 142(2), 213–228. https://doi.org/10.1097/GIM.0b013e3181faa0d2 28981371 10.5858/arpa.2016-0621-OA

[mgg3381-bib-0002] Baptista, J. , Mercer, C. , Prigmore, E. , Gribble, S. M. , Carter, N. P. , Maloney, V. , … Crolla, J. A. (2008). Breakpoint mapping and array CGH in translocations: Comparison of a phenotypically normal and an abnormal cohort. American Journal of Human Genetics, 82(4), 927–936. https://doi.org/10.1016/j.ajhg.2008.02.012 18371933 10.1016/j.ajhg.2008.02.012 PMC2427237

[mgg3381-bib-0003] Caramins, M. C. , Saville, I. , Shakeshaft, R. , Mullan, G. L. , Miller, B. , Yip, M. Y. , & Buckley, M. F. (2011). A comparison of molecular and cytogenetics techniques for the diagnosis of pregnancy loss. Genetics in Medicine, 13(1), 46–51. 21102343 10.1097/GIM.0b013e3181faa0d2

[mgg3381-bib-0004] Dhillon, R. K. , Hillman, S. C. , Morris, R. K. , McMullan, D. , Williams, D. , Comarasamy, A. , & Kilby, M. D. (2014). Additional information from chromosomal microarray analysis (CMA) over conventional karyotyping when diagnosing chromosomal abnormalities in miscarriage: A systematic review and meta‐analysis. British Journal of Obstetrics and Gynecology, 121(1), 11–21. https://doi.org/10.1111/1471-0528.12382 10.1111/1471-0528.12382 23859082

[mgg3381-bib-0005] Fisher, R. A. , Tommasi, A. , Short, D. , Kaur, B. , Seckl, M. J. , & Sebire, N. J. (2014). Clinical utility of selective molecular genotyping for diagnosis of partial hydatidiform mole; a retrospective study from a regional trophoblastic disease unit. Journal of Clinical Pathology, 67(11), 980–984. https://doi.org/10.1136/jclinpath-2014-202517 25078332 10.1136/jclinpath-2014-202517

[mgg3381-bib-0006] Furtado, L. V. , Paxton, C. N. , Jama, M. A. , Tripp, S. R. , Wilson, A. R. , Lyon, E. , … Geiersback, K. B. (2013). Diagnostic utility of microsatellite genotyping for molar pregnancy testing. Archives of Pathology and Laboratory Medicine, 137(1), 55–63. https://doi.org/10.5858/arpa.2012-0047-OA 23276175 10.5858/arpa.2012-0047-OA

[mgg3381-bib-0007] Lathi, R. B. , Gustin, S. L. F. , Keller, J. , Maisenbacher, M. K. , Siqurjonsson, S. , Tao, R. , & Demko, Z. (2014). Reliability of 46, XX results on miscarriage specimens: A review of 1,222 first‐trimester miscarriage specimens. Fertility and Sterility, 101(1), 178–182. https://doi.org/10.1016/j.fertnstert.2013.09.031 24182409 10.1016/j.fertnstert.2013.09.031

[mgg3381-bib-0008] McGowan‐Jordan J., Simons A., & Schmid M. (Eds.) (2016). ISCN (2016): An international system for human cytogenomic nomenclature. Basel: S. Karger.

[mgg3381-bib-0009] Murugappan, G. , Gustinl, S. , & Lathi, R. B. (2014). Separation of miscarriage tissue from maternal decidua for chromosome analysis. Fertility and Sterility, 102(4), e9–e10. https://doi.org/10.1016/j.fertnstert.2014.07.006 25150388 10.1016/j.fertnstert.2014.07.006

[mgg3381-bib-0010] Pinkel, D. , Straume, T. , & Gray, J. W. (1986). Cytogenetic analysis using quantitative, high‐sensitivity, fluorescence hybridization. Proceedings of the National Academy of Sciences of the United States of America, 83(9), 2934–2938. https://doi.org/10.1073/pnas.83.9.2934 3458254 10.1073/pnas.83.9.2934 PMC323421

[mgg3381-bib-0011] Rosenfeld, J. A. , Tucker, M. E. , Escobar, L. F. , Neill, N. J. , Torchia, B. S. , McDaniel, L. D. , … Chitayat, D. (2015). Diagnostic utility of microarray testing in pregnancy loss. Ultrasound in Obstetrics and Gynecology, 46(4), 478–486. https://doi.org/10.1002/uog.14866 25846569 10.1002/uog.14866

[mgg3381-bib-0012] Sahoo, T. , Dzidic, N. , Strecker, M. N. , Commander, S. , Travis, M. K. , Doherty, C. ,… Hovanes, K. (2017). Comprehensive genetic analysis of pregnancy loss by chromosomal microarrays: Outcomes, benefits, and challenges. Genetics in Medicine, 19(1), 83–89. 27337029 10.1038/gim.2016.69

[mgg3381-bib-0013] Shearer, B. M. , Thorland, E. C. , Carlson, A. W. , Jalal, S. M. , & Ketterling, R. P. (2011). Reflex fluorescent in situ hybridization testing for unsuccessful product of conception cultures: A retrospective analysis of 5555 samples attempted by conventional cytogenetics and fluorescent in situ hybridization. Genetics in Medicine, 13(6), 545–552. https://doi.org/10.1097/GIM.0b013e31820c685b 21415758 10.1097/GIM.0b013e31820c685b

[mgg3381-bib-0014] Tabet, A. C. , Verloes, A. , Pilorge, M. , Delaby, E. , Delorme, R. , Nygren, G. ,… Betancur, C. (2015). Complex nature of apparently balanced chromosomal rearrangements in patients with autism spectrum disorder. Molecular Autism, 6(19), 1–14. 25844147 10.1186/s13229-015-0015-2 PMC4384291

[mgg3381-bib-0015] Trask, B. , & Pinkel, D. (1990). Fluorescence in situ hybridization with DNA probes In Darzynkiewicz Z. & Crissman H. A. (Eds.), Methods in cell biology (pp. 383–400). San Diego, CA: Academic Press. 10.1016/s0091-679x(08)60542-7 2084477

[mgg3381-bib-0016] Van Dyke, D. L. , & Wiktor, A. (2002). Clinical cytogenetics In McClatchey K. D. (Ed.), Clinical laboratory medicine: Self‐assessment and review, 2nd edn (pp. 589–635). Philadelphia, PA: Lippincott Williams & Wilkins.

[mgg3381-bib-0017] Wang, Y. , Cheng, Q. , Meng, L. , Luo, C. , Hu, H. , Zhang, J. , … Xu, Z. (2017). Clinical application of SNP array analysis in first‐trimester pregnancy loss: A prospective study. Clinical Genetics, 91(6), 849–858. https://doi.org/10.1111/cge.12926 27883173 10.1111/cge.12926

[mgg3381-bib-0018] Wiktor, A. E. , Van Dyke, D. L. , Stupca, P. J. , Ketterling, R. P. , Thorland, E. C. , Shearer, B. M. , … Dewald, G. W. (2006). Preclinical validation of fluorescence in situ hybridization assays for clinical practice. Genetics in Medicine, 8(1), 16–23. 16418595 10.1097/01.gim.0000195645.00446.61

